# The relationship between psychological resilience and employability among higher vocational college students: the chain mediating effects of perceived social support and career decision-making self-efficacy

**DOI:** 10.3389/fpsyg.2025.1707655

**Published:** 2026-01-13

**Authors:** Ye Xiong, Jijiang Yu, Hewen Wu

**Affiliations:** 1 Faculty of Education, Shaanxi Normal University, Xi’an, China; 2 Jiangxi Vocational College of Industry & Engineering, Pingxiang, China

**Keywords:** career decision-making self-efficacy, employability, higher vocational college, perceived social support, psychological resilience

## Abstract

**Introduction:**

The global youth is facing challenges of large-scale job-seeking pressure and structural employment contradictions. Employability is a key factor in alleviating employment pressure. Grounded in the conservation of resources theory, this study aims to explore the relationship between psychological resilience and employability among higher vocational college students, with a specific focus on the chain mediating effects of perceived social support and career decision-making self-efficacy.

**Methods:**

A total of 1,709 students from five higher vocational colleges with industrial features in Jiangxi Province, China, were surveyed using the Psychological Resilience Scale, Perceived Social Support Scale, Career Decision-Making Self-Efficacy Scale, and Employability Scale. The relationships among these variables were tested using PROCESS plugin for SPSS version 27.0.

**Results:**

The results indicated that (1) there were significant positive correlations among all variables; (2) psychological resilience had a significant positive impact on employability; and (3) psychological resilience could affect employability through three indirect pathways, namely, the independent mediation of perceived social support and career decision-making self-efficacy, and their chain mediation.

**Discussion:**

It is suggested that higher vocational colleges should integrate psychological resilience training into the curriculum, establish a multi-stakeholder collaborative support network, and implement experiential career guidance programs. This study provides an empirical support for improving employability among higher vocational college students.

## 1 Introduction

The contemporary labor market is characterized by rapid technological advancement, economic fluctuations, and increasing employment instability (Jabeen et al., 2025). In this highly uncertain employment environment, graduates worldwide are encountering unprecedented challenges in their transition from education to workplace. In 2023, the World Economic Forum released “the Future of Jobs Report 2023,” which indicated that by 2027, 23% of global jobs would undergo structural changes driven by automation technology, highlighting the urgency and necessity of skills transformation (World Economic Forum, 2023). Concurrently, the supply pressure on the labor market continues to mount. In 2025, the Ministry of Education of the People’s Republic of China issued a notice on “Implementing Employment and Entrepreneurship Support for the 2025 Graduates,” which showed that the number of college graduates in China reached 12.22 million, an increase of 430,000 over the previous year, with further growth anticipated in subsequent years (Ministry of Education of the People’s Republic of China, 2025). More notably, youth employment is confronted with particularly severe challenges. In 2024, the International Labour Organization released a report “World Employment and Social Outlook: Trends 2024,” which pointed out that the global youth unemployment rate had risen to 13.2%, nearly triple that of adults (International Labour Organization, 2024). These figures indicate that global youth are facing the challenges of large-scale job-seeking pressure and structural employment contradictions. Against this backdrop, higher vocational colleges, as main institutions for cultivating high-level skilled and application-oriented talents, should take enhancing students’ employability as a key link to alleviate employment pressure (Zhou et al., 2023).

Employability is a multidimensional and comprehensive concept. It does not merely refer to individuals’ ability to secure a job, but rather emphasizes their potential to maintain employment, switch jobs, and achieve sustainable career development in the labor market (Hillage and Pollard, 1998; Heijde and Van Der Heijden, 2006). According to Fugate et al. (2004), employability encompasses multiple dimensions, including career identity, personal adaptability, social capital, and human capital. It reflects the comprehensive competence of workers in adapting to the dynamic labor market through the organic integration of knowledge, skills, attitudes, and psychological capital. Against the current employment landscape, repeated setbacks and continuous pressure during the job-seeking process can easily trigger psychological distress among graduates, such as anxiety, self-doubt, occupational burnout, and even depression (Jang and Lee, 2025; Chen and Zheng, 2024). These issues not only undermine their job-seeking motivation but may also lead to more serious mental health crises. This highlights the urgent need for higher vocational colleges to broaden their talent educational requirements, not only to equip students with solid technical skills but also to consciously foster positive psychological resources, which enable them to cope with adversity and maintain mental wellbeing. Therefore, from the perspective of positive psychology, exploring the internal psychological motivation mechanisms for enhancing employability is particularly important.

Conservation of resources theory provides a robust theoretical framework for understanding this dynamic mechanism. Initially proposed by Hobfoll (1989), the theory posits that the fundamental motivation of individual behavior lies in striving to avoid resource loss and pursue resource gain. It defines resources as “anything that individual values, including objects, conditions, energy, or personal characteristics” and introduces the principle of “loss aversion,” which states that the psychological distress caused by resource loss is far greater than the satisfaction brought by an equivalent amount of resource gain (Hobfoll, 1989; Hobfoll and Shirom, 2000). The job-seeking process is inherently a stressful situation fraught with potential resource loss (e.g., time, energy, self-confidence). It is worth noting that exploring how resources promote positive developmental outcomes has become a cutting-edge paradigm in youth research. For example, studies have revealed that external environmental resources (e.g., urban green spaces) can enhance the quality of life of young people by regulating psychological mechanisms such as loneliness (Ortiz Diaz and Abbas, 2025). This paradigm suggests that when examining the role of internal psychological resources (e.g., psychological resilience) in employability, we also need to systematically analyze the psychological processes and social mechanisms through which they exert their effects. Psychological resilience, as a positive psychological resource, refers to a psychological quality that enables individuals to mobilize protective resources (both internal and external) to achieve positive adaptation when facing adversities, crisis events, and similar situations (Masten, 2001). Previous study has shown that individuals with high levels of psychological resilience demonstrate higher job performance regardless of changes in labor market conditions (Shen Z. -M.et al., 2024), which indicates that psychological resilience is a stable predictor of enhancing employability. While existing studies have focused on the impact of psychological capital on employability (Khalid et al., 2025; Karimi et al., 2025), the underlying mechanisms through which psychological resilience acts on employability have not yet been sufficiently elucidated.

Based on conservation of resources theory, psychological resilience may enhance employability by activating and integrating other key resources. This resource gain process is first manifested in the effective utilization of external support systems. Perceived social support, as a crucial external resource, can provide individuals with emotional, informational, and instrumental support. Critically, the subjective perception of such support has a more significant impact on individuals’ resource evaluation than its objective existence (Zimet et al., 1988), thereby initiating the resource gain cycle. Subsequently, these activated external resources must be transformed into internal decision-making motivation. Career decision-making self-efficacy, as a key personal trait resource, plays a central mediating role in this process by bolstering confidence in confronting career decision-making challenges (Betz et al., 1996). Essentially, it functions as the mechanism that converts psychological resources (e.g., resilience) and social resources (e.g., support) into motivation for job-seeking behaviors. For instance, individuals with high psychological resilience are more likely to proactively seek social support. In turn, the perceived support can strengthen their career decision-making confidence, thereby forming a “resource gain cycle.” This chain mediation pathway is particularly significant for higher vocational college students, as they not only face pressure from skill mismatch but may also experience self-doubt due to perceived academic disadvantages (Sugimura et al., 2025), which can accelerate resource depletion.

Therefore, this study, based on conservation of resources theory, takes students from five higher vocational colleges with industrial features in Jiangxi Province, China, aiming to explore the relationship between psychological resilience and employability among higher vocational college students, with a specific focus on the chain mediation effects of perceived social support and career decision-making self-efficacy. This research can not only provide a theoretical basis for mental health crisis intervention but also offer empirical support for improving employability for higher vocational college students.

## 2 Literature review and research hypotheses

### 2.1 Psychological resilience and employability

According to conservation of resources theory, individuals are motivated to acquire, retain, and protect the resources they value, including time, energy, social capital, and psychological assets (Hobfoll, 1989). In the uncertain employment environment, these resources are vulnerable to threats or even loss. Psychological resilience can not only mitigate resource loss but also facilitate resource gain (Hobfoll and Shirom, 2000; Wang et al., 2023). Compared with other graduates, higher vocational college students often have relatively weaker initial human capital and social capital, making their resource reservoirs more susceptible to the impact of job-seeking setbacks (Li et al., 2024). This may deplete key psychological resources such as self-confidence and optimism, thereby potentially triggering a “resource loss spiral” (Hobfoll and Shirom, 2000). Resource loss may lead to further resource depletion, ultimately resulting in job-seeking burnout, career disorientation, or even withdrawal from the labor market. In contrast, students with high levels of psychological resilience can effectively interrupt this vicious cycle. Specifically, psychological resilience can act as a “resource buffer,” alleviating the impact of employment anxiety on psychological resources through positive emotion regulation and cognitive reappraisal (Yang et al., 2025), protecting individuals’ resource reservoirs from severe depletion. Additionally, psychological resilience demonstrates positive resource investment behaviors (Hobfoll and Shirom, 2000), driving individuals to proactively enhance career adaptability, search for employment opportunities, and pursue employment goals with stronger motivation, firmer determination, and more sustained perseverance (Fang and Xu, 2025). These behaviors help individuals accumulate human capital and social capital, thereby initiating a “resource gain spiral” (Hobfoll and Shirom, 2000). Existing research indicates that positive psychological resources are key factors in predicting individuals’ diverse career development outcomes. This conclusion is not only supported by studies in the employability field (Tam et al., 2024), but has also been validated in other important developmental domains such as social entrepreneurship (Shabbir et al., 2025). Based on this reasoning, the following hypothesis is proposed:

*H1*: Psychological resilience positively predicts employability among higher vocational college students.

### 2.2 The mediating effect of perceived social support

Social support refers to the subjective experience and objective assistance that individuals receive from various aspects of society, serving as a critical indicator of the quality of their social relationships (Zimet et al., 1988). Perceived social support reflects individuals’ expectations and subjective feelings about the potential support from others, which has been proven to be a key variable in predicting perceived employability (Oliveira et al., 2025), occupational adaptability (Rençber and Paşaoğlu Baş, 2023), and maintaining mental health (Huang et al., 2023). In the highly competitive labor market, higher vocational college students face significant career development challenges and are more prone to experience employment anxiety and negative career expectations. According to conservation of resources theory, perceived social support provides critical external resources for individuals to deal with employment stress (Hobfoll, 2002), making them confident that they can obtain support from others in difficult times (Zimet et al., 1988). Empirical studies have shown that perceived support from parents and friends contributes to the improvement of job-related skills, practical experience, and work performance (Li et al., 2025). During corporate internships, support from mentors has also been found to significantly enhance students’ confidence in obtaining employment (Shen P. et al., 2024). Additionally, peer support facilitates the development of subject knowledge, interpersonal skills, and transferable skills (Despotovic et al., 2024). Notably, whether individuals can effectively perceive and utilize social support is closely related to their level of psychological resilience (Min and Rong, 2025). Individuals with higher psychological resilience are more likely to proactively seek and accept external support in the face of setbacks. They also tend to interpret supportive messages from family, friends, and others in a positive manner, which collectively contributes to the enhancement of employability. Based on this, the following hypothesis is proposed:

*H2*: Perceived social support mediates the relationship between psychological resilience and employability among higher vocational college students.

### 2.3 The mediating effect of career decision-making self-efficacy

Career decision-making self-efficacy refers to individuals’ confidence and positive evaluation of their ability to make career decision, which helps form a clear self-concept and occupational role cognition (Betz et al., 1996). According to conservation of resources theory, career decision-making self-efficacy is regarded as a critical personal trait resource that significantly influences individuals’ cognitive and behavioral performance, including career cognition, job-seeking behavior, work attitude, and occupational activities (Hobfoll, 2002; Ahmad and Nasir, 2023). Individuals with higher career decision-making self-efficacy possess a clearer understanding of their career goals and values, exhibit stronger intrinsic motivation and behavioral initiative, actively confront career challenges, and continue to make efforts, thereby achieving more positive career development outcomes. Existing studies have shown that career decision-making self-efficacy significantly promotes career adaptability (Karimi et al., 2024), job satisfaction (Fris et al., 2025), career intention (Chowdhury et al., 2025), and job-seeking behavior (Xiong et al., 2025), and is positively correlated with employability (Zhou et al., 2023). Furthermore, psychological capital has been shown to positively influence career decision-making self-efficacy (Zhou et al., 2024). As a core component of psychological capital, psychological resilience is likely to enhance individuals’ employability by improving their career decision-making self-efficacy. Based on the above analysis, the following hypothesis is proposed:

*H3*: Career decision-making self-efficacy mediates the relationship between psychological resilience and employability among higher vocational college students.

### 2.4 The chain mediating effect of perceived social support and career decision-making self-efficacy

Previous research has shown that psychological resilience influences employability, and this relationship may be mediated by external resources such as perceived social support, or by personal trait resources such as career decision-making self-efficacy. According to conservation of resources theory, perceived social support represents a practical pathway, providing tangible support and risk buffering that enables individuals to pursue career goals in a more sustained and secure manner (Zimet et al., 1988; Hobfoll, 2002). In contrast, career decision-making self-efficacy belongs to a self-driven pathway, relating to individuals’ confidence and motivation to achieve vocational objectives (Betz et al., 1996; Hobfoll, 2002). These two factors address the issues of “possibility” and “feasibility” in employment activities, respectively, from the two dimensions of external resources and personal trait resources. Together, they motivate individuals to initiate and persist in job-seeking behaviors, thereby effectively achieving career expectations.

Furthermore, according to conservation of resources theory, different types of resources can interact, transform, and reinforce each other, giving rise to “resource gain spirals” (Hobfoll, 2002). As a key external resource, perceived social support can be converted into personal trait resources (Zimet et al., 1988; Hobfoll, 2002). Specifically, when individuals perceive a high level of social support, it strengthens their confidence and motivation in their career decision-making abilities (Zhou et al., 2024), thereby promoting more proactive career behaviors and the development of their capabilities (Zhou et al., 2023; Xiong et al., 2025; Karimi et al., 2024). This resource conversion pathway has been further validated in youth behavior research in the digital era. For example, studies have found that the information support and community belongingness that young people gain on social media can effectively enhance their online efficacy, thereby driving their political participation behaviors (Santos et al., 2025). This provides important cross-domain insights into understanding how contemporary vocational college students convert online and offline social support into career decision-making confidence and ultimately improve their employability. Studies have found that psychological resilience is a crucial antecedent variable influencing the level of perceived social support (Fang and Xu, 2025; Naz et al., 2024). Based on this, the following hypothesis is proposed:

*H4*: Perceived social support and career decision-making self-efficacy play a chain mediating role between psychological resilience and employability among higher vocational college students.

**Figure 1:**
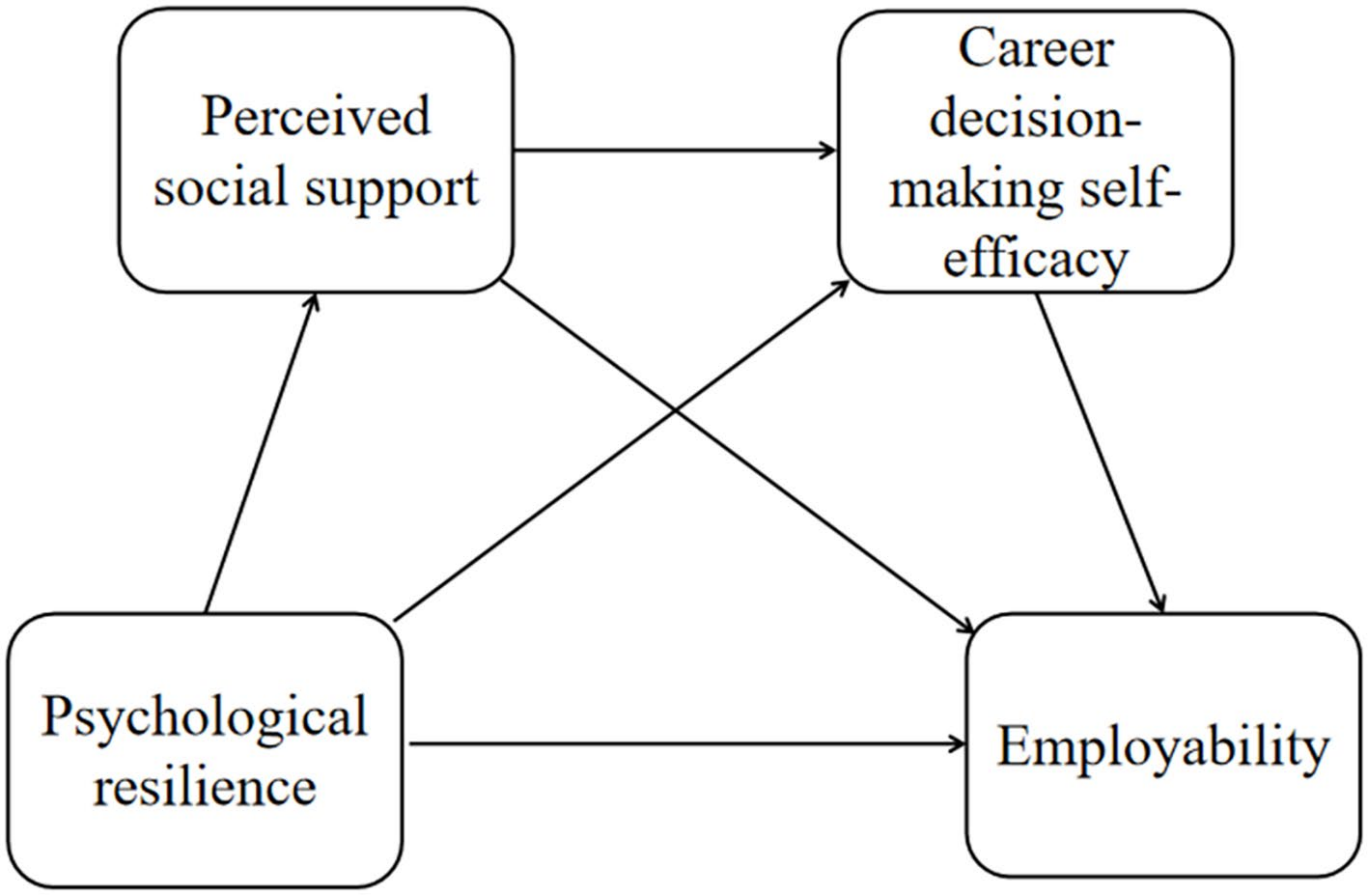
Hypothetical model.

Grounded in the above literature review and research hypotheses, this study constructs a hypothetical model, as shown in Figure 1.

## 3 Research methods

### 3.1 Participants

This study employed a convenience sampling method to recruit participants from five higher vocational colleges with industrial features in Jiangxi Province, China, including Jiangxi Vocational College of Industry & Engineering, Jiangxi Application Engineering Vocational College, Jiangxi Metallurgical Vocational and Technical College, Jiangxi Environmental Engineering Vocational College, and Jiangxi Biotech Vocational College. These colleges rank in the middle tier within the province, none of which is selected for the “Jiangxi Double High-Level Plan.” They share similar educational levels and student enrollment scales. The inclusion criteria for participants were as follows: (1) full-time freshman to junior students; (2) voluntary participation; (3) ability to independently comprehend and complete the questionnaire; and (4) completion of demographic information and the questionnaire in more than 3 min. Exclusion criteria included: (1) obvious patterned responses (e.g., consecutively selecting the same option); (2) part-time or deregistered students. After preliminary communication with instructors to confirm their willingness to assist in distributing the questionnaire, the survey was conducted via the Questionnaire Star platform from April 29 to May 10, 2025, with anonymity and data confidentiality ensured throughout the process. A total of 1,763 questionnaires were collected. After excluding 54 invalid responses, 1,709 valid questionnaires were retained, resulting in an effective response rate of 96.94%. Among the participants, there are 928 males (54.3%) and 781 females (45.7%); 409 freshmen (23.9%), 740 sophomores (43.3%), and 560 juniors (32.8%); 718 participants from non-STEM major (42%) and 991 participants from STEM major (58%); 164 only child (9.6%) and 1,545 non-only child (90.4%); and 148 participants from urban areas (8.7%), 316 participants from counties (18.5%), and 1,245 participants from rural areas (72.8%).

### 3.2 Measurement of variables

#### 3.2.1 Psychological resilience

The Psychological Resilience Scale developed by Connor and Davidson (2003) was adopted. It consists of 25 items, measuring three dimensions: hardiness, self-enhancement, and optimism. A Likert 5-point rating scale is used for scoring (1 = “strongly disagree,” 5 = “strongly agree”), where a higher total score indicates a higher level of psychological resilience. The overall Cronbach’s *α* coefficient of the scale in this study was 0.967 (Watson et al., 1988), and the *α* coefficients for the three dimensions were 0.951 (hardiness), 0.911 (self-enhancement), and 0.733 (optimism), respectively. The construct validity indices were as follows: *χ*^2^/df = 2.384, NFI = 0.987, CFI = 0.993, GFI = 0.980, TLI = 0.987, AGFI = 0.964, and RMSEA = 0.028.

#### 3.2.2 Perceived social support

The Scale of Perceived Social Support developed by Zimet et al. (1988) was adopted. It consists of 12 items divided into three dimensions: perceived family support, perceived friends support, and perceived support from significant others (referring to support from teachers, relatives, classmates, etc.). A Likert 7-point rating scale is used for scoring (1 = “strongly disagree,” 7 = “strongly agree”), with higher total scores indicating a greater level of perceived social support. The overall Cronbach’s *α* coefficient of the scale was 0.966 in this study (Watson et al., 1988), and the *α* coefficients for the respective dimensions were 0.937 (perceived family support), 0.938 (perceived friends support), and 0.927 (perceived other support). The construct validity indices were as follows: *χ*^2^/df = 1.835, NFI = 0.998, CFI = 0.999, GFI = 0.995, TLI = 0.998, AGFI = 0.986, and RMSEA = 0.022.

#### 3.2.3 Career decision-making self-efficacy

The Career Decision-Making Self-Efficacy Scale revised by Chen et al. (2022) was adopted, based on the instruments developed by Betz et al. (1996). It consists of 11 items divided into five dimensions: self-evaluation, information collection, goal setting, plan formulation, and problem-solving. A Likert 5-point rating scale is used for scoring (1 = “Strongly Unconfident,” 5 = “Strongly Confident”). The higher the score, the stronger the career decision-making self-efficacy. In the present study, the overall Cronbach’s *α* coefficient of the scale was 0.970 (Watson et al., 1988), and the *α* coefficients for the respective dimensions were 0.888 (self-evaluation), 0.9 (information collection), 0.868 (goal setting), 0.911 (plan formulation), and 0.908 (problem-solving). The construct validity indices were as follows: *χ*^2^/df = 2.368, NFI = 0.997, CFI = 0.999, GFI = 0.994, TLI = 0.996, AGFI = 0.983, and RMSEA = 0.028.

#### 3.2.4 Employability

The Employability Scale for higher vocational college students revised by Chen (2024), based on the Career EDGE model proposed by Lorraine Dacre Pool and Sewell (2007). It comprises 26 items covering five dimensions: emotion management and self-regulation, academic performance and learning skills, career development learning, problem-solving ability, and work and life experience. A Likert 5-point rating scale is used for scoring (1 = “Strongly disagree,” 5 = “Strongly agree”) where a higher score indicates stronger employability. In the present study, the overall Cronbach’s α coefficient of the scale was 0.974 (Watson et al., 1988), and the α coefficients for the dimensions in sequence were 0.940 (emotion management and self-regulation), 0.904 (academic performance and learning skills), 0.916 (career development learning), 0.856 (problem-solving ability), and 0.836 (work and life experience). The construct validity indices were as follows: *χ*^2^/df = 2.375, NFI = 0.991, CFI = 0.995, GFI = 0.983, TLI = 0.989, AGFI = 0.964, and RMSEA = 0.028.

### 3.3 Data processing

The data were analyzed using SPSS 27.0 for the common method bias test, reliability and validity test, descriptive statistics, independent samples *t*-tests, one-way ANOVA, and correlation analysis among variables. Mediation analysis was performed using Model 6 from the SPSS macro program PROCESS 4.0 plugin.

## 4 Results

### 4.1 Common method bias test

In this study, all four measurement instruments were used to assess the same group of participants, and all data were collected via self-report. To control for common method bias, the anonymity, authenticity and confidentiality of the assessment were emphasized in the instructions before the test began. During the data analysis phase, Harman single-factor test was used to conduct an exploratory factor analysis on all scale items (Podsakoff et al., 2003). The results showed that there were six factors with eigenvalues greater than 1, and the variance explained by the largest common factor was 18.769%, which was far below the critical threshold of 40% (Chen et al., 2025). Based on this, it can be concluded that there is no significant issue of common method bias in this study.

### 4.2 Reliability and validity test

To examine the reliability and validity of the scale, this study used SPSS 27.0 to calculate KMO, composite reliability (CR), and other indicators for psychological resilience, perceived social support, career decision-making self-efficacy, and employability. The results showed that the KMO values of each research variable were greater than 0.7, and the CR values were all greater than 0.7, indicating that these variables meet the conditions for high reliability (Fornell and Larcker, 1981). The factor loadings of all variables were greater than 0.7, exceeding the critical value of 0.5, which suggested good validity of the variables (Fornell and Larcker, 1981). The AVE values of each research variable were all greater than 0.5, indicating good discriminant validity (Bollen, 1987). These statistical indicators and their results show that the variables involved in this study meet the statistical requirements and are suitable for further analysis.

### 4.3 Differences in demographic variables among higher vocational college students

The participants were divided into different groups based on demographic variables, such as gender, major, grade, only child and home location. Independent samples *t*-test and one-way ANOVA were used to analyze the differences in psychological resilience, perceived social support, career decision-making self-efficacy, and employability across these groups. The specific statistical results are presented in Table 1.

**Table 1:** Analysis of differences in demographic variables.

Variables	Index	Psychological resilience	Perceived social support	Career decision-making self-efficacy	Employability
Gender	Male	3.79 ± 0.67	5.33 ± 1.16	3.80 ± 0.74	3.75 ± 0.70
	Female	3.59 ± 0.59	5.16 ± 1.01	3.63 ± 0.64	3.59 ± 0.59
	*t*	6.39^***^	3.21^***^	5.23^***^	5.14^***^
Major	Non-STEM	3.68 ± 0.65	5.25 ± 1.09	3.72 ± 0.70	3.67 ± 0.66
	STEM	3.71 ± 0.63	5.25 ± 1.10	3.72 ± 0.70	3.68 ± 0.66
	*t*	−0.84	−0.01	−0.01	−0.15
Grade	Freshman	3.63 ± 0.60	5.17 ± 1.10	3.71 ± 0.65	3.58 ± 0.62
	Sophomore	3.72 ± 0.66	5.30 ± 1.09	3.70 ± 0.73	3.69 ± 0.67
	Junior	3.72 ± 0.64	5.25 ± 1.09	3.76 ± 0.70	3.73 ± 0.66
	F	3.24^*^	0.18	0.27	7.15^***^
Only child	Yes	3.79 ± 0.70	5.43 ± 1.18	3.77 ± 0.80	3.73 ± 0.72
	No	3.69 ± 0.63	5.23 ± 1.09	3.72 ± 0.69	3.67 ± 0.65
	*t*	1.75	2.2^*^	0.85	1.05
Home location	Urban	3.90 ± 0.71	5.72 ± 1.01	3.95 ± 0.75	3.90 ± 0.72
	County	3.71 ± 0.67	5.25 ± 1.12	3.73 ± 0.72	3.69 ± 0.66
	Rural	3.67 ± 0.62	5.20 ± 1.01	3.69 ± 0.69	3.65 ± 0.65
	F	8.77^***^	15.34^***^	9.01^***^	9.75^***^

*N* = 1709, ^*^p < 0.05, ^**^p < 0.01, ^***^p < 0.001.

The results showed that higher vocational college students of different genders exhibited significant differences in psychological resilience (*t* = 6.39, *p* < 0.001), perceived social support (*t* = 3.21, *p* < 0.001), career decision-making self-efficacy (*t* = 5.23, *p* < 0.001), and employability (*t* = 5.14, *p* < 0.001), with males achieving significantly higher scores than females across all variables. Significant differences were also observed across different grade levels in psychological resilience (*F* = 3.24, *p* < 0.05) and employability (*F* = 7.15, *p* < 0.001). *Post-hoc* tests revealed that freshmen had significantly higher scores on both variables than sophomores and juniors, while no significant difference was found between sophomores and juniors. A significant difference in perceived social support was observed between only child and non-only child (*t* = 2.2, *p* < 0.05), with only child scoring higher. Students from different home location display significant differences in psychological resilience (*F* = 8.77, *p* < 0.001), perceived social support (*F* = 15.34, *p* < 0.001), career decision-making self-efficacy (*F* = 9.01, *p* < 0.001), and employability (*F* = 9.75, *p* < 0.001). *Post-hoc* comparisons indicate that students from cities had significantly higher scores on all variables than those from counties and rural areas, and students from counties had significantly higher scores than those from rural areas. Furthermore, no significant differences were observed among students from different majors across all research variables. Based on the above findings, gender, grade, only child, and home location were included as covariates in the subsequent analysis to control for their potential impact on the dependent variable.

### 4.4 Descriptive statistics and correlation analysis

Descriptive statistics and correlation analysis were conducted for all research variables, with results shown in Table 2. Psychological resilience, perceived social support, career decision-making self-efficacy, and employability were all significantly correlated. Specifically, psychological resilience was significantly positively correlated with perceived social support (*r* = 0.72, *p* < 0.001), career decision-making self-efficacy (*r* = 0.79, *p* < 0.001), and employability (*r* = 0.80, *p* < 0.001). Similarly, perceived social support was significantly positively linked to both career decision-making self-efficacy (*r* = 0.71, *p* < 0.001) and employability (*r* = 0.69, *p* < 0.001). A particularly positive correlation was observed between career decision-making self-efficacy and employability (*r* = 0.88, *p* < 0.001).

**Table 2:** Descriptive statistics and correlation analysis.

Research variables	M	SD	1	2	3	4
1 Psychological resilience	3.70	0.64	1			
2 Perceived social support	5.25	1.10	0.72^***^	1		
3 Career decision-making self-efficacy	3.72	0.70	0.79^***^	0.71^***^	1	
4 Employability	3.68	0.66	0.80^***^	0.69^***^	0.88^***^	1

*N* = 1709, ^*^p < 0.05, ^**^p < 0.01, ^***^p < 0.001.

### 4.5 Test of the chain mediation effect

The chain mediation effect was tested using Model 6 from the SPSS macro program PROCESS 4.0 plugin. In this analysis, psychological resilience was set as the independent variable, employability as the dependent variable, perceived social support and career decision-making self-efficacy as chain mediating variables, and gender, grade, only child, and home location were included as covariates in the regression model. The hypothetical model was tested using the sequential test method and bootstrap method (with 5,000 bootstrap samples) for analyzing the mediating effects.

**Figure 2:**
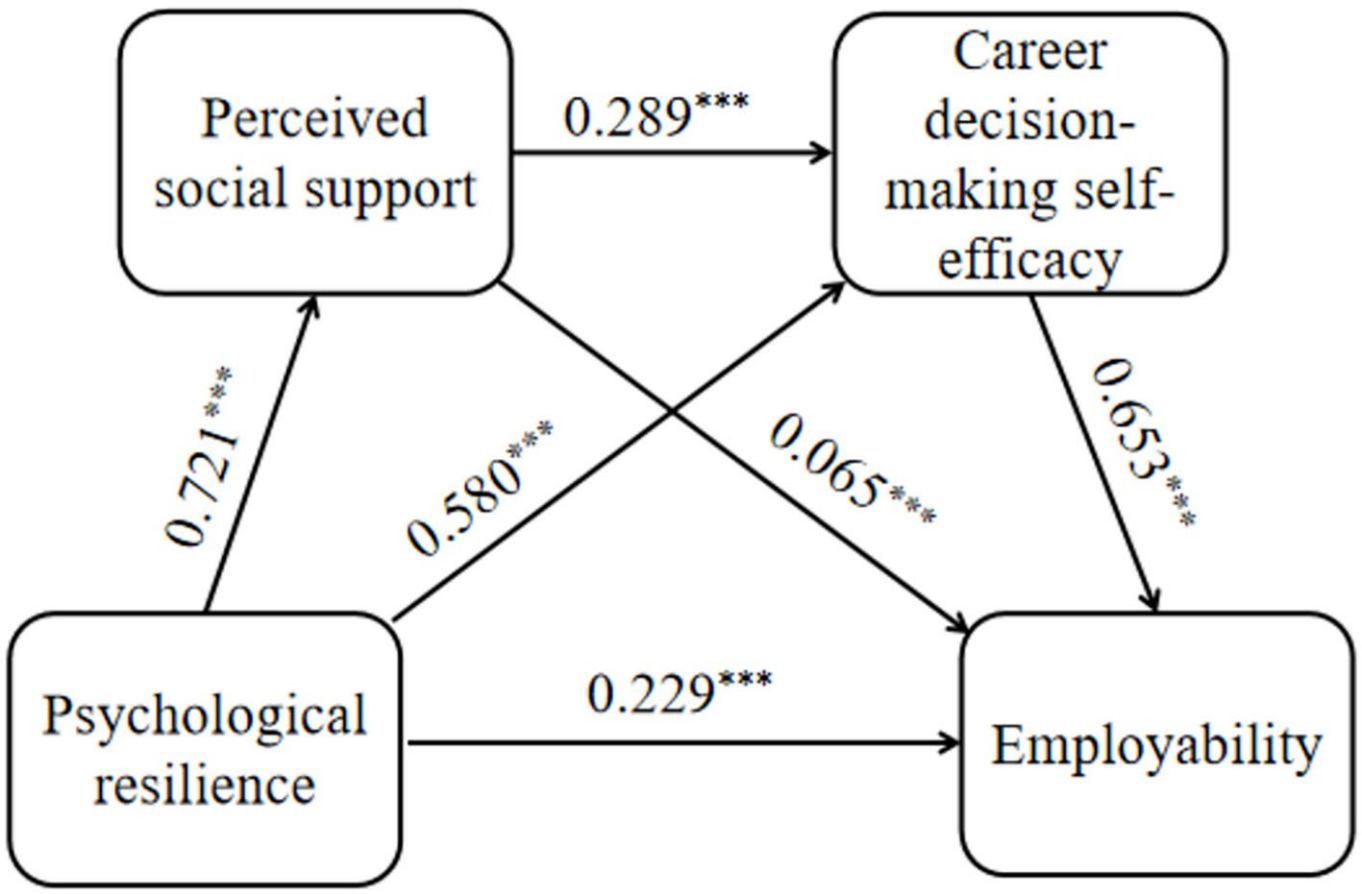
The chain mediating effect of perceived social and career decision-making self-efficacy between psychological resilience and employability. *N* = 1709, ^*^p < 0.05, ^**^p < 0.01, ^***^p < 0.001.

The results showed (see Table 3 and Figure 2) that all pathways in the model were significant, and the variance explained by each model reached a significant level (*F* = 372.160, 558.509, 1036.147, *p* < 0.001), indicating that the model fit indices met the criteria. Specifically, psychological resilience significantly positively predicted perceived social support (*β* = 0.721, *p* < 0.001), career decision-making self-efficacy (*β* = 0.580, *p* < 0.001), and employability (*β* = 0.229, *p* < 0.001). Perceived social support also had a significant positive predictive effect on career decision-making self-efficacy (*β* = 0.289, *p* < 0.001) and employability (*β* = 0.065, *p* < 0.001). Additionally, career decision-making self-efficacy significantly positively predicted employability (*β* = 0.653, *p* < 0.001).

**Table 3:** Regression analysis of the relationship between variables in the chain mediation model.

Model	Outcome	Predictor	*R*	*R* ^2^	*F*	*β*	*t*	LLCI	ULCI
Model 1	②	①	0.723	0.522	372.160^***^	0.721	42.273^***^	1.175	1.289
Model 2	③	①	0.814	0.663	558.509^***^	0.580	28.265^***^	0.589	0.677
		②				0.289	14.182^***^	0.159	0.210
Model 3	④	①	0.900	0.810	1036.147^***^	0.229	12.259^***^	0.198	0.273
		②				0.065	4.031^***^	0.020	0.058
		③				0.653	35.873^***^	0.582	0.649

*N* = 1709, ① Psychological resilience, ② perceived social support, ③ career decision-making self-efficacy, ④ employability. ^*^p < 0.05, ^**^p < 0.01, ^***^p < 0.001.

The results of mediation effect test (see Table 4) indicated that the direct effect of psychological resilience on employability was 0.236, accounting for 28.993% of the total effect. The Bootstrap 95% confidence interval for this direct effect did not include zero, indicating a statistically significant direct effect, namely, psychological resilience exerted a significant positive impact on employability among higher vocational college students, supporting Hypothesis 1. Perceived social support and career decision-making self-efficacy partially mediated the relationship between psychological resilience and employability, with a total indirect effect value of 0.578, accounting for 71.007% of the total effect. Psychological resilience influenced employability through three significant mediating pathways, as evidenced by Bootstrap 95% confidence intervals that excluded zero for all pathways. The specific pathways and their effect sizes were as follows: (1) Psychological resilience → perceived social support → employability, with an effect value of 0.048, accounting for 5.897% of the total effect, indicating that perceived social support played a significant mediating role between psychological resilience and employability among higher vocational college students, thus Hypothesis 2 is supported; (2) Psychological resilience → career decision-making self-efficacy → employability, with an effect value of 0.390, accounting for 47.911% of the total effect, showing that career decision-making self-efficacy had a significant mediating effect between psychological resilience and employability among higher vocational college students, thereby validating Hypothesis 3; (3) Psychological resilience → perceived social support → career decision-making self-efficacy → employability, with an effect value of 0.140, accounting for 17.199% of the total effect, showing that perceived social support and career decision-making self-efficacy had a significant chain mediating effect between psychological resilience and employability among higher vocational college students, thus verifying Hypothesis 4.

**Table 4:** Analysis of mediating effects of perceived social support and career decision-making self-efficacy.

Effect	Pathway	*β*	*β*%	95%CI	
				Lower	Upper
Total effect		0.814	100%	0.784	0.844
Direct effect		0.236	28.993%	0.198	0.273
Total indirect effect		0.578	71.007%	0.532	0.622
Indirect effect pathway 1	① → ② → ④	0.048	5.897%	0.018	0.079
Indirect effect pathway 2	① → ③ → ④	0.390	47.911%	0.339	0.439
Indirect effect pathway 3	① → ② → ③ → ④	0.140	17.199%	0.106	0.175

*N* = 1709, ① psychological resilience, ② perceived social support, ③ career decision-making self-efficacy, ④ employability.

## 5 Discussion

### 5.1 Analysis of differences in demographic variables among higher vocational college students

The results of this study indicate that males scored significantly higher than females on all variables, including psychological resilience, perceived social support, career decision-making self-efficacy, and employability. This consistent pattern may stem from a combination of factors across multiple levels. Firstly, according to social role theory, traditional gender roles often expect males to exhibit instrumental traits such as stress tolerance, independence, and self-confidence (Eagly, 2013; Dos Santos, 2022). This may lead males to report higher scores in self-assessments of psychological resilience and career decision-making self-efficacy. Secondly, regarding the differences in perceived social support, a possible explanation is that the scale measures not only emotional support but also instrumental and informational support (Zimet et al., 1988). Males may be more likely to perceive practical support related to career development within their peer networks, or have greater confidence in accessing support. This is associated with the socialization process wherein males are encouraged to utilize networks to obtain resources (Hamdani et al., 2023; Wang et al., 2024; Dong et al., 2024; Beroíza-Valenzuela, 2025). Additionally, the advantage in resource access brought about by macro-level social structures may enable male students to develop a broader perception of resource availability (Chen et al., 2024; Callander et al., 2025), thereby systematically influencing their self-reported scores.

Regarding grade differences, freshmen scored significantly lower than senior students in terms of psychological resilience and employability. This is consistent with the general laws of psychological development (Lent and Brown, 2013). Notably, however, no significant differences were observed between sophomore and junior students. This may reveal a non-linear trajectory in psychological and career development (Davis, 2006). During the transition from freshman to sophomore year, students experience a critical leap in adaptability and career awareness. After entering sophomore year, development may enter a relatively stable phase (Schaller, 2005). Another explanation stems from the specific context of higher vocational education, where major skill training and concentrated internships are mostly arranged in the sophomore year. This may have brought about a key improvement in students’ psychological resources and employment confidence. In the junior year, the focus may shift towards job application, resulting in no significant improvement in level, hence the lack of significant score difference between sophomore and junior students (Nunley et al., 2016).

Only child scored significantly higher in perceived social support than non-only child. This difference may be associated with resource allocation and the density of attention received during growth. According to the resource dilution hypothesis, non-only child need to share limited family resources with their siblings (De Leeuw et al., 2022), resulting in relatively weaker actual access to support and subjective perception of support (Hosany and Hamilton, 2023). In terms of home location, urban students scored significantly higher in all variables than those from counties and rural areas. The essence of this difference may be attributed to disparities in socioeconomic status and uneven resource allocation (Róbert, 2021). Students in urban areas typically enjoy more favorable economic conditions, cultural capital, and social networks (Strømme and Wiborg, 2024), thereby gaining more learning opportunities, broader career perspectives, and a stronger foundation for employability. Beyond the above, there were no significant differences across students from different majors on any of the variables. This may be due to the homogenization trend in higher education talent cultivation and the increasing demand for general competencies in the labor market (George and Paul, 2024; Iqbal et al., 2023).

### 5.2 The effect of psychological resilience on employability

This study indicated that psychological resilience can significantly positively predict employability among higher vocational college students, which supports hypothesis H1. This finding is consistent with existing research, further confirming that psychological resilience helps students effectively regulate stress, enhance adaptability, and achieve sustainable occupational competitive advantages in the employment process (Fang and Xu, 2025; Tam et al., 2024). As an important central province, Jiangxi is in a critical phase of industrial transformation and upgrading, accompanied by notable structural contradictions in its labor market (Fan and Shang, 2025). Students from higher vocational colleges with industrial features are key sources of skilled talents, and their employment quality and career development status are directly linked to the sustainable development and innovative competitiveness of the local economy. However, this group of students is confronted with multiple practical challenges. Jiangxi Province has a relatively weak regional economic foundation, and there are certain biases in talent policies and social perceptions. Moreover, the overall talent cultivation quality of higher vocational colleges still has room for improvement. Additionally, the widespread application of new technologies, such as automation and artificial intelligence, has exerted an impact on the employment structure, manifesting as reduced job opportunities, skill mismatch, limited career advancement channels, and relatively low wage levels (Nakamura, 2023; Agrawal et al., 2023). In addition, many of these students are employed in specific industries or frontline production, their work environment often involve high-intensity and high-adaptability demands, placing particularly high requirements on psychological resources. A lack of psychological resilience may easily result in a decline in their employability and even trigger the phenomenon of short-term turnover. Therefore, this research finding provides empirical evidence for enhancing employability through psychological resilience interventions, and also offer a directional reference for employment guidance work in higher vocational colleges.

### 5.3 The mediating effect of perceived social support

This study revealed that perceived social support plays a significant mediating effect between psychological resilience and employability among higher vocational college students, supporting hypothesis H2. This conclusion aligns with previous research, which emphasizes the importance of both psychological resources and social support resources for constructing sustainable employability (Tam et al., 2024; Jabeen et al., 2025). Employability is not merely a collection of knowledge and skills, but rather a dynamic psycho-social process in which individuals interact with external support systems to achieve career identity construction and value realization. The employment process is highly dynamic, influenced by a range of internal and external factors, such as labor market fluctuations, technological changes, personal skill structures, and career expectations, leading to uncertainty in employment outcomes. According to goal setting theory, individuals can make dynamic adjustments based on the discrepancy between expectation and actual outcome (Locke and Latham, 2002). When actual outcome falls below expectation, individuals may proactively seek and mobilize social support to bridge the gap and adjust strategies. When actual outcome exceeds expectation, social support can also assist individuals in setting more challenging career goals, driving them to achieve higher-level career development. However, not all students will proactively mobilize social support to adjust their job-seeking behaviors when facing employment pressure and setbacks. For students who are not good at perceiving and utilizing social support, teachers should emphasize enhancing their psychological resilience to indirectly improve their ability to perceive and utilize social support, thereby promoting the overall development of their employability.

### 5.4 The mediating effect of career decision-making self-efficacy

This study indicated that career decision-making self-efficacy plays a crucial mediating role between psychological resilience and the employability among higher vocational college students, thereby supporting hypothesis H3. On the one hand, psychological resilience positively predicts career decision-making self-efficacy, which is consistent with existing research (Zhou et al., 2024). Students with higher levels of psychological resilience tend to demonstrate greater self-confidence and problem-solving abilities when confronted with decision-making tasks, such as career selection, information gathering, and future planning, all of which contribute to the development of higher-level career decision-making self-efficacy. On the other hand, career decision-making self-efficacy exerts a significant positive predictive effect on the employability among higher vocational college students, which is also supported by prior studies (Zhou et al., 2023). Students with stronger career decision-making self-efficacy are more likely to actively engage in career exploration, clarify their career goals, and effectively implement job-seeking behaviors, thus demonstrating more comprehensive capabilities in the context of employment competition. These findings can be explained through self-efficacy theory (De Leeuw et al., 2022). Specifically, psychological resilience enhances students’ career decision-making self-efficacy by providing emotional regulation support and accumulating successful experiences, which in turn drives them to adopt more goal-oriented behaviors, ultimately leading to improved employability.

It is important to emphasize that the mediating path effect of career decision-making self-efficacy accounts for 47.911%. According to conservation of resources theory, psychological resilience is a positive psychological resource, and perceived social support is a key external resource (Hobfoll, 2002), and both are static resources. However, for higher vocational college students, they not only face the pressure of skill mismatch in the job market but also often develop self-doubt due to their cognitive disadvantage in academic background (Sugimura et al., 2025). In the repeated setbacks of the job-seeking process, they are prone to experience confidence depletion (Jang and Lee, 2025; Chen and Zheng, 2024). Career decision-making self-efficacy acts as an important conversion hub in this process (Hobfoll, 2002), transforming these static resources into a series of goal-oriented and sustained job-seeking behaviors, including active information collection, assessment of skill gaps, and execution of learning plans. In other words, students with strong career decision-making self-efficacy can dynamically convert static psychological and social capital into a sustainable path for enhancing employment competitiveness.

### 5.5 The chain mediating effect of perceived social support and career decision-making self-efficacy

This study revealed that psychological resilience exerts a significant positive impact on employability among higher vocational college students through the chain mediating effect of perceived social support and career decision-making self-efficacy, thus supporting hypothesis H4. This research conclusion can be explained from the perspective of self-determination theory. Perceived social support effectively stimulates individuals’ intrinsic motivation by satisfying their three basic psychological needs, including autonomy, competence, and relatedness (Ryan and Deci, 2000). In the context of higher vocational education in China, influenced by cultural traditions such as respect for teachers and value of learning and relationship-oriented culture (Markus and Kitayama, 1991), students often attribute deeper career significance to support received from teachers and enterprise mentors. This support not only conveys knowledge and skills but is also closely linked to industry skill recognition, professional qualification access, and future career identity (Fugate et al., 2004). As a result, it can more effectively meet students’ psychological needs and serve as a key catalyst for stimulating their intrinsic motivation for career development (Ryan and Deci, 2000). Specifically, compared with individuals with low perceived social support, students with high perceived social support are more likely to develop a higher level of career decision-making self-efficacy. They tend to set “performance-approach goals” oriented toward pursuing success, rather than “performance-avoidance goals” motivated by avoiding failure. Deci and Ryan’s (2000) research further indicates that when the goals set by individuals are both challenging and autonomous, and can satisfy the sense of competence, their performance in task execution and level of sustained engagement are significantly better than those of individuals who set avoidance goals. Therefore, students with higher level of psychological resilience are better able to perceive and utilize social support. The key role of this lies in helping them internalize external supportive resources as autonomous career development motivation, thereby enhancing their career decision-making self-efficacy and ultimately improving their employability.

## 6 Strengths, limitations and future directions

The strengths of this study are as follows. Firstly, previous studies have primarily focused on the direct effect of psychological factors on employability. In contrast, this study constructs a chain mediation model based on conservation of resources theory. From a positive psychology perspective, it validates the internal mechanism through which psychological resilience affects employability among higher vocational students by influencing perceived social support and career decision-making self-efficacy, providing empirical support for the formation of employability. Secondly, this study focuses on students from higher vocational colleges with industrial features, addressing the current context of technological changes and employment uncertainty. The findings have practical significance for aligning higher vocational education with industry needs and conducting psychological resilience curriculum. Finally, this study provides clear guidelines for students growth and development, guiding students to consciously cultivate psychological resilience, actively seek and perceive social support resources, enhance confidence and decision-making abilities in career decision-making, thereby systematically strengthening their employability, ultimately promoting sustainable career development.

The limitations and future directions of this study are as follows. Firstly, all samples in this study were collected from vocational colleges of similar types within the same province. While this design enhances the internal validity and depth of the research on the specific group, it also limits the external validity of the conclusions. Future research should conduct cross-sample validation across different economic development regions, different types of institutions, and regular undergraduate universities to verify the generalizability of the model and explore potential boundary conditions. Secondly, although this study has verified the chain mediating effect of perceived social support on career decision self-efficacy, there is still room for expansion in the research model. On the one hand, there may be other mediating variables that have not been included. Future studies can integrate more variables for exploration, especially in the context of the digital age, where emerging factors such as technology anxiety and human−computer collaboration literacy may have a significant impact on employability. On the other hand, there may be more complex dynamic interactive relationships between variables. Future research can investigate the bidirectional or reciprocal relationship between perceived social support and career decision self-efficacy to more comprehensively and systematically reveal the pathways of employability formation. Finally, this study adopted a cross-sectional design with self-report scales. Although statistical methods were used to control for some biases, it is difficult to rigorously infer causal relationships between variables, and outcome variables such as employability lack objective behavioral indicators. Future research could adopt a longitudinal tracking design, collecting data at multiple time points to more clearly reveal the dynamic influence pathways of psychological resilience, social support, and career decision self-efficacy on employability. Additionally, attempts could be made to combine behavioral indicators for multi-method validation, such as actual job-seeking behavior records, internship unit evaluations, and simulated interview performance, to enhance the robustness of research conclusions and causal inference power.

## 7 Conclusions and recommendation

The conclusions of this study demonstrate significant positive correlations among psychological resilience, perceived social support, career decision-making self-efficacy, and employability among higher vocational college students. Furthermore, psychological resilience exhibits a significant direct and positive impact on employability among higher vocational college students. It also enhances employability among higher vocational college students through three distinct indirect pathways: the independent mediating effects of perceived social support and career decision-making self-efficacy; and the chain mediating effects of perceived social support and career decision-making self-efficacy.

To improve employability among higher vocational college students, we recommend the following: (1) Incorporate psychological resilience training into higher vocational curriculum, with an emphasize on developing practical skills (such as stress management, emotional regulation, and adaptive coping strategies) rather than purely theoretical knowledge. (2) Establish a multi-stakeholder collaborative support network involving college, enterprise, family, and peer aimed at improving the ability of perceive and utilize social support of higher vocational college students. (3) Implement experiential career guidance programs, such as simulated interviews, project-based challenges, and mentoring sessions with industry professionals, to enhance career decision-making self-efficacy of higher vocational college students.

## Data Availability

The raw data supporting the conclusions of this article will be made available by the authors, without undue reservation.
